# A cross‐sectional study on the relationship between meditation training and emotional intelligence in women

**DOI:** 10.1002/nop2.715

**Published:** 2020-11-27

**Authors:** Min‐Kyu Sung, Na Hyun Ha, Ul Soon Lee, Hyun‐Jeong Yang

**Affiliations:** ^1^ Korea Institute of Brain Science Seoul Korea; ^2^ Department of Brain‐based Emotion Coaching Global Cyber University Seoul Korea; ^3^ Department of Brain Education Convergence Global Cyber University Seoul Korea; ^4^ Department of Integrative Health Care University of Brain Education Cheonan Korea

**Keywords:** emotional intelligence, meditation, melatonin, middle aged, sleep, women

## Abstract

**Aim:**

We aimed to reveal the relationship of meditation with emotional intelligence (EI), sleep quality and melatonin level.

**Design:**

A cross‐sectional study.

**Methods:**

Our current research was performed on middle‐aged women. EI scale, Pittsburgh Sleep Quality Index (PSQI) and night‐time saliva melatonin were measured for 65 participants including 33 meditators and 32 controls.

**Results:**

The meditation group showed a significantly higher EI score than the control group. In the regression analysis between EI and age, only the meditation group showed a significant positive correlation. The Pearson correlation analysis among all participants revealed a significant negative correlation between PSQI and EI. There was no significant group difference in the melatonin and PSQI.

## 1 INTRODUCTION

Emotional intelligence (EI) is defined as an ability that recognizes emotions of oneself and of others, distinguishes different emotions and expresses and regulates the emotions (Mayer and Salovey, 1997). EI has been conceptualized into ability EI, which measures actual level of emotional intelligent performance and trait EI, which reflects typical emotional intelligent functioning (Pérez et al., 2005). According to the meta‐analysis, trait EI is more strongly related to health than ability EI (Martins et al., 2010). Researches about relationships between EI and mental health indicators such as depression and anxiety suggest that higher EI is linked to better mental health (Martins et al., 2010; Schutte et al., 2007). Moreover, such a trend is verified beyond cultures (Bhullar et al., 2012). In the relationship between EI and age, EI level‐age graph draws an inverted‐U shape with age 32–44 as the peak for EI level (Cabello et al., 2016). Thus, EI of women decreases along with age after midlife. The EI reflects physical and psychological health (Zeidner et al., 2012). Researchers have suggested that EI is linked to depression. In a study of the older people, higher EI was associated with beneficial effects on the depressive symptoms (Lloyd et al., 2012). In addition, EI is a predictive indicator of depression and disruptive behaviour for adolescents (Davis and Humphrey, 2012). Major depression is higher in women than men by 1.7‐fold (Baxter et al., 2014; Cyranowski et al., 2000; Ford & Erlinger, 2004; Whiteford et al., 2013). Its higher prevalence in women than men is commonly observed regardless of countries, suggesting the difference is based on biological factors rather than socioeconomical factors (Rai et al., 2013). Women experience dramatic hormonal changes as menopause progresses and risks of depression increases during perimenopausal transition (Cohen et al., 2006). Therefore, especially emotional management of this period is important for mental health of women.

## 2 BACKGROUND

When the brains of the long‐term brainwave vibration (BWV) meditation group (mixed gender group, mean age: 24 years) and the control group were compared using functional magnetic resonance imaging, greater functional connectivity was observed in the medial prefrontal cortex in the BWV group when compared with the control group (Jang et al., 2011). The medial prefrontal cortex area is known to regulate emotional processing by suppressing amygdala (Arnsten, 2009). When the brain structures of the two groups were compared, higher fractional anisotropy values and greater cortical thicknesses were observed in the BWV group in the medial prefrontal cortex, suggesting increased thickness of the white matter and the grey matter at the site (Kang et al., 2013). The BWV meditators exhibited a significantly elevated EI compared with the control patients (Choi et al., 2018), indicating results consistent with the functional and the structural changes of the brain. However, there have been no reports about the relationship of the meditation method with EI in middle‐aged women who experience emotional alterations by hormonal changes.

Although depressive symptoms are not directly related to hormonal changes, disruptions in emotional management and depressive symptoms are common during menopause through various indirect pathways (Berent‐Spillson et al., 2017). Therefore, emotional regulations are critical issues especially for middle‐aged women compared with other ages. In the current study, we aimed to understand the relationship between meditation training and EI as well as sleep and salivary melatonin secretion in women of midlife.

## 3 DESIGN

The present cross‐sectional study belongs to the project aimed at finding any potential association of meditation with adult health states. The research was performed between September 2018 and December 2018 at the university located in Republic of Korea. Participants were recruited over a month through posting in the facility, social networking service and phone notice at the meditation training centres and the universities. The present research included healthy adult women who were able to perform the physical and mental activities of the regular meditation training level. The meditation group included women who performed Brain Education Sangdahnjeon Meditation (BESM) at least once a week for 6 months. BESM training consists of two steps: dynamic BWV training (Bowden et al., 2012; Bowden et al., 2014; Jang et al., 2018; Lee et al., 2015) and static focused attention training (Lee, 2005). Participants filled the self‐report forms and collected the saliva at home by the guideline. For the present research, samples of 66 participants were initially intended for the analysis. However, one person was excluded due to non‐completion of the self‐report. Thus, 65 samples (meditation = 33, control = 32) were analysed.

## 4 METHOD

### 4.1 Self‐reports

The EI scale is a 20‐item questionnaire designed to measure trait EI in Korean adults. It has a three‐factor structure: emotional recognition/understanding, emotional facilitation and emotional regulation (Lee & Kwak, 2012). Higher scores indicate better EI. Pittsburgh Sleep Quality Index (PSQI) is a self‐report questionnaire that measures sleep quality and disturbances during a 1‐month period. It consists of seven component scores: subjective sleep quality, sleep latency, sleep duration, habitual sleep efficiency, sleep disturbances, use of sleeping medication and daytime dysfunction (Buysse et al., 1989). Lower scores indicate better sleep quality.

### 4.2 Salivary melatonin assessments

#### 4.2.1 Saliva sample collection

The participants were instructed not to undergo dental treatment during 24 hr prior to sample collection, as it may affect salivary hormone levels. Additionally, they were asked about their oral health including presence of wounds to check for sample quality. Consumption of bananas, chocolate, alcohol and medications; smoking; and excessive exercise were prohibited on the day of sample collection. After having a regular dinner, drinks other than water and food intake during the night were also prohibited on the day of sample collection. The participants were asked to maintain their regular sleep pattern on the day of collection and rinse their mouths thoroughly before sleep. Samples were collected at 3 hr after the regular bedtime immediately after waking up to an alarm under minimum dim light conditions (<10 Lux), as the half‐life of melatonin is short (approximately 10 min) and night light acutely inhibits melatonin synthesis (Middleton, 2013). After collecting the sample (1 ml) in a sterile high‐quality polypropylene tube, it was capped firmly and immediately stored at −20°C in a freezer. On the next day, it was delivered to the laboratory in a frozen state and stored at −80°C in the laboratory freezer until the analysis. The participants were asked to submit the checklist including the sample collection date and time with their samples. Samples were labelled only by a number code, to blind the researchers for analysis.

#### 4.2.2 Enzyme‐linked immunosorbent assay

For analysis, the saliva samples were returned to room temperature, mixed and centrifuged at 1,500 g for 15 min. The supernatant was then used for the analysis. Salivary melatonin levels were measured by enzyme‐linked immunosorbent assay (ELISA) according to the manufacturer's instructions (Salivary Melatonin Enzyme Immunoassay Kit, 1–3402, Salimetrics, State College, PA). All samples were run in duplicate. The intra‐assay (repeatability) and inter‐assay (reproducibility) coefficients of variation were 4.08 and 12.56%, respectively.

### 4.3 Analysis

An optimal total sample size of *N* = 52 required to perform Student's *t* test (effect size *d* = 0.8 with a significance level set at α = 0.05, power 1 – β = 0.80) was calculated using the G‐Power software application. Other analyses were performed using the SPSS, version 25. Group differences in the number of postmenopausal participants and religion of participants were tested using chi‐square tests. *T* tests were used to investigate the differences of age, education; to compare the self‐report results and the melatonin level between two groups. Regression analysis or correlation analysis was used to examine the relation among the investigated factors. Statistical significance was based on two‐sided statistical tests and was evaluated at a 0.05 level of significance.

### 4.4 Ethics

The research was performed at the university in Republic of Korea. Participants were recruited at the local meditation training centres and local universities. The study was approved by the Ethics Committee of the local university. The research was carried out in accordance with The Code of Ethics of the World Medical Association (Declaration of Helsinki) for experiments involving humans and according to the STROBE guidelines for cross‐sectional studies. All the participants provided written informed consents. The authors have no competing interests to report.

## 5 RESULTS

We analysed the data of 65 healthy women including 33 long‐term meditators and 32 meditation‐naïve controls. No statistically significant differences were observed in age (meditation versus. control: 48.67 ± 6.30 versus. 46.72 ± 9.25 years, mean ± standard deviation, *p* = .323, *t* test), menopausal state (postmenopause in meditation versus. control: 16 versus. 11, *p* = .248, chi‐squared test) and education periods (meditation versus. control: 16.30 ± 3.32 versus. 16.09 ± 3.20 years, *p* = .797, *t* test) between the groups. In addition, there were no differences in the distribution of religion (non‐religious/Buddhism/Protestantism/Catholicism/other in meditation versus. control: 19/4/2/1/7 versus. 15/7/5/4/1, *p* = .065, chi‐squared test). In the meditation group, total training period, daily practice time and daily practice frequencies were 10.32 ± 6.24 years, 40.00 ± 33.68 min/day and 2.24 ± 1.90 times/day, respectively.

**Figure 1:**
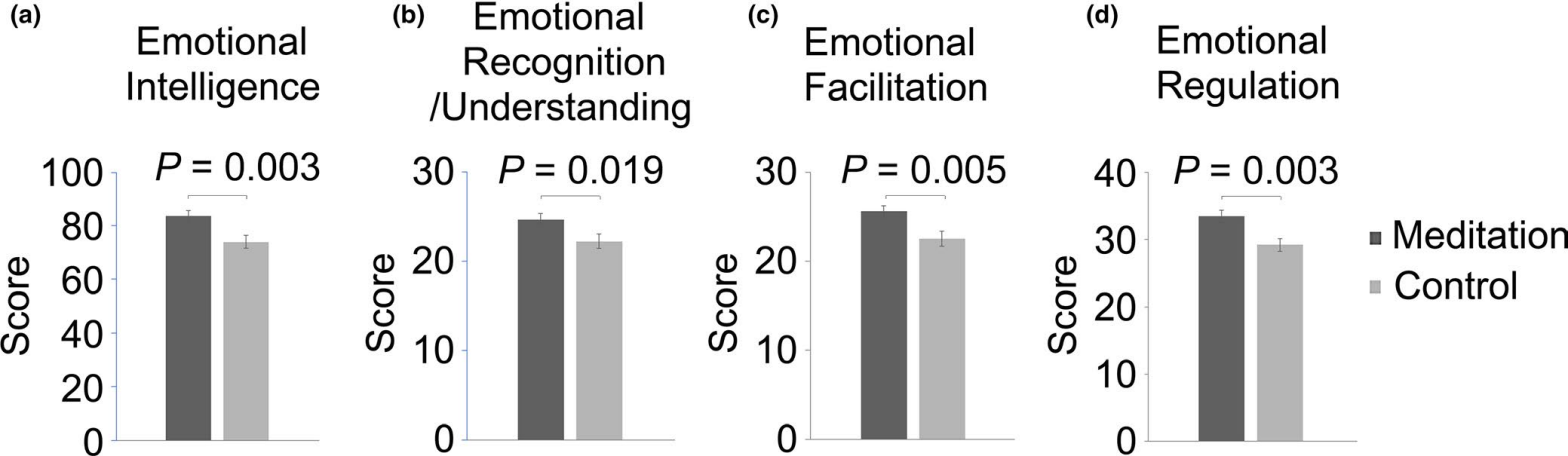
Score comparisons of emotional intelligence and its subscales between the meditation group and the control group. Graph and error bars indicate mean ± standard error. *p*‐values were calculated using Student's *t* test

To study the relationship between meditation and EI in middle‐aged women, we compared the EI score of the meditation and the control group. EI is composed of emotional recognition/understanding, emotional facilitation and emotional regulation. In the total EI score and in all the sub‐items, the meditation group showed significantly higher scores when compared with the control group (meditation versus. control, total score, 83.61 ± 11.41 versus. 73.97 ± 13.67, *p* = .003; emotional recognition, 24.64 ± 3.62 versus. 22.22 ± 4.46, *p* = .019; emotional facilitation, 25.55 ± 3.58 versus. 22.53 ± 4.69, *p* = .005; emotional regulation, 33.42 ± 5.32 versus. 29.22 ± 5.52, mean ± standard deviation, *p* = .003, *t* test) (Figure 1). The results indicate that the meditation training is related to EI in middle‐aged women.

**Figure 2:**
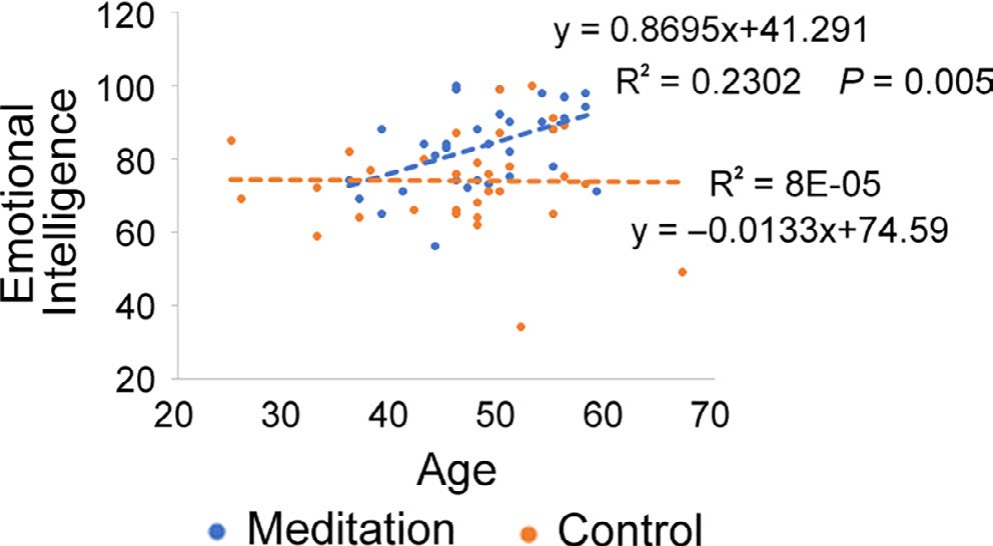
Relationship between emotional intelligence and age in the meditation and the control group. Regression analysis between emotional intelligence and age in the meditation group (blue dots and dotted line) and the control group (orange dots and dotted line). Indicated *p*‐value was calculated using regression analysis in the meditation group

According to the previous research (Cabello et al., 2016), trait EI was not changed or even reduced depending on the sub‐components since 40s in women. Consistent with this, the control group showed no changes in EI scores according to age in the current study (Figure 2). However, interestingly, we found a significant positive correlation between EI and age, only in the meditation group (*p* = .005, Regression analysis, Figure 2). There were no significant correlations between age and other investigated factors; melatonin level or PSQI in both groups.

**Figure 3:**
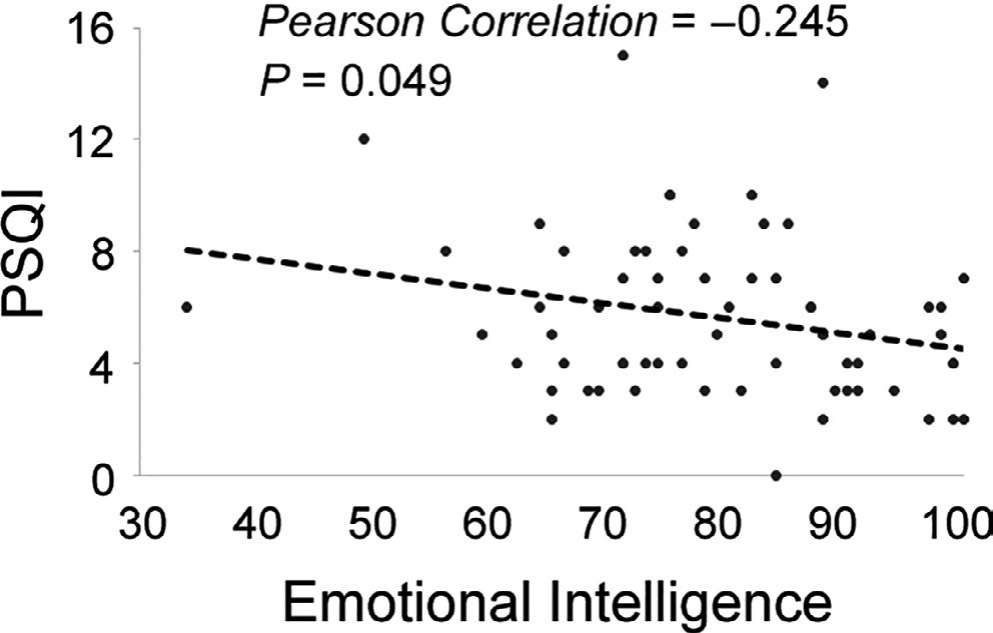
Correlation analysis between the Pittsburgh Sleep Quality Index and emotional intelligence among all participants. *p*‐value was calculated using Pearson's correlation analysis

To figure out the correlation among the investigated factors in general, we performed correlation analysis on the data of all participants. We found a significant negative correlation between EI and PSQI (*p* = .049, Pearson correlation, Figure 3). No significant correlations were found between melatonin level and PSQI (*p* = .748) or between melatonin level and EI (*p* = .418).

**Figure 4:**
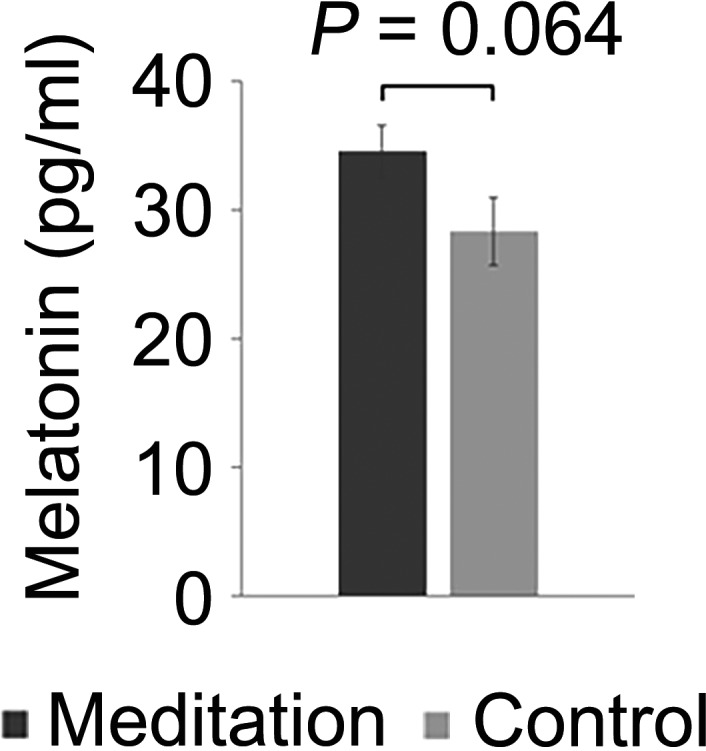
Comparison of salivary melatonin concentration between the meditation group and the control group. Graph and error bars indicate mean ± standard error. *p*‐value was calculated using Student's *t* test

To compare the level of melatonin between the meditation and the control group, night‐time salivary melatonin level was measured by ELISA. The meditation group showed a tendency of increase in the melatonin level when compared with the control group (34.49 *SD* 2.02 versus. 28.2 (*SD* 2.62) pg/ml, *p* = .064, *t* test, Figure 4).

To measure the quality of sleep in the two groups, we used the PSQI questionnaires. Although no significant differences or tendencies were observed, the mean total PSQI (meditation versus. control: 5.33 (*SD* 2.78) versus. 5.94 (*SD* 3.06), Table 1) and most of the sub‐items including sleep quality, sleep latency, sleep efficiency and sleep medication exhibited lower scores in the meditation group when compared with the control group.

**Table 1:** The Pittsburgh sleep quality index

Group	Meditation	Control
*N*	33	32
Total score	5.33 ± 2.78	5.94 ± 3.06
Q1: Sleep quality	0.97 ± 0.64	1.09 ± 0.59
Q2: Sleep latency	0.82 ± 0.85	1.13 ± 0.94
Q3: Sleep duration	0.85 ± 0.83	0.75 ± 0.80
Q4: Sleep efficiency	0.18 ± 0.47	0.28 ± 0.68
Q5: Sleep disturbance	1.09 ± 0.58	1.16 ± 0.45
Q6: Sleep medication	0.09 ± 0.53	0.22 ± 0.75
Q7: Daytime dysfunction	1.33 ± 0.96	1.31 ± 0.86

Note

Indicated values are mean ± standard deviation of total and individual item scores.

## 6 DISCUSSION

In the current study, the BESM meditation group exhibited higher EI scores compared with the control group (Figure 1). One previous research about EI on meditators of BWV, which was used as a dynamic meditation part of the BESM, showed higher EI in the meditator than in the control group (Choi et al., 2018). In the Choi's study, the mean age of the participants was about 26 years old and 36% of the participants was men. Emotional state changes together with age. Especially, middle‐aged women, as menopause starts, depression and anxiety increase (Vivian‐Taylor & Hickey, 2014). However, it has not been reported about the relation of BWV or BESM with emotional intelligence which affects emotional regulation, for this specific age of women. In the current study, we recruited the long‐term women meditators of BESM training which includes BWV meditation, and the mean age of the participants was about 47 years old. The meditation group showed a better EI compared with the control group not only in young adults (Choi et al., 2018), but also in middle‐aged women (Figure 1), suggesting the strong relationship between the meditation training method and EI.

Interestingly, though there was no correlation between age and EI in the control group, long‐term meditators exhibited a higher EI with an increase in age (Figure 2). In women, EI decreases with age after midlife (Cabello et al., 2016). Therefore, our results suggest that meditation training may reverse this phenomenon. Long‐term BWV training, a dynamic component of BESM used in the current study, activates the medial prefrontal cortex (J. H. Jang et al., 2011) and thickens the grey and the white matter of the medial prefrontal cortex (Kang et al., 2013), suggesting improvement in emotional regulation. These meditation‐induced functional and structural brain changes support our finding of improved EI after the long‐term meditation training.

Meta‐analysis indicated the importance of the link between trait EI and mental health (Martins et al., 2010). In the study which examined 855 participants, EI was suggested to be a predictive indicator for mental health (Fernandez‐Abascal & Martin‐Diaz, 2015). Therefore, the relationship between the meditation training and EI will provide the evidences which can be used in disease prevention related to mental health, by further researches to reveal the causal relations.

Among all participants, there was a negative correlation between PSQI and EI (Figure 3). In other words, it suggests a correlation between better sleep quality and higher EI. Our finding is consistent with previous reports: sleep deprivation is related to lower scores in total EI (Killgore et al., 2008) and fewer sleep complaints correlate with higher EI (Emert et al., 2017).

In the current study, no correlation was observed between physiological night‐time salivary melatonin level and PSQI and between melatonin level and EI. In the previous research (Toffol et al., 2014), no relationship was observed between physiological night‐time serum melatonin level and sleep quality, depression level and quality of life, which is consistent with our findings.

## 7 CONCLUSIONS

In the present study, the long‐term meditators exhibited a significant increase in EI compared with the controls. Interestingly, EI increased with age only in the meditation group, but not in the control group. In the correlation analysis targeting all participants, there was a negative correlation between EI and PSQI. Sleep quality is an important factor which affects quality of life. The negative correlation between EI and PSQI indicates meditation practice which increases EI level would enhance quality of life by affecting sleep quality. Therefore, in terms of nursing practice, the complementary use of meditation training would help to increase the quality of life of patients. Although we found potential relationship of meditation with the above‐mentioned factors through the current study, a longitudinal randomized controlled trial is required to know the causal relationships. This will contribute to the application of the meditation training to the clinical use as a complementary method for future health care. In addition, while the current study included middle‐aged women, the relations of the meditation training with EI for populations of other age range (such as adolescents or elders) or men are expected to be answered for same purpose.

## CONFLICT OF INTEREST

The authors declare that there is no conflict of interest.

## AUTHOR CONTRIBUTIONS

Min‐Kyu Sung: Data curation, Validation, Formal analysis, Visualization, Writing. Ul Soon Lee: Conceptualization. Na Hyun Ha: Funding acquisition, Investigation. Hyun‐Jeong Yang: Funding acquisition, Conceptualization, Methodology, Project administration, Writing, Supervision.

## ETHICAL STATEMENTS

Following includes detailed information which was not included in the “Participants” section of the main document due to the anonymous policy. The research was performed at University of Brain Education, Cheonan, Republic of Korea. Participants were recruited at the Brain Training Centers (Daejeon, Pusan, Seoul), Global Cyber University (Cheonan, Seoul) and University of Brain Education (Cheonan). The study was approved by the Ethics Committee of the University of Brain Education. The authors have no competing interests to report.

## Data Availability

The authors declare that the data supporting the findings of this study are available within the article.
